# Evaluating the Effects of Perioperative Ketorolac Use on Uncemented Total Hip Arthroplasty Outcomes

**DOI:** 10.3390/jcm14144956

**Published:** 2025-07-13

**Authors:** Mehul M. Mittal, David Edwards, Antonia F. Chen, Varatharaj Mounasamy, Senthil N. Sambandam

**Affiliations:** 1 Department of Orthopaedics, University of Texas Southwestern Medical Center, Dallas, TX 75390, USA; david.edwards@phhs.org (D.E.); antonia.chen@utsouthwestern.edu (A.F.C.); varatharaj.mounasamy@utsouthwestern.edu (V.M.); senthil.sambandam@utsouthwestern.edu (S.N.S.); 2 Surgical Service, Dallas Veterans Affairs Medical Center, Dallas, TX 75216, USA

**Keywords:** total hip arthroplasty, ketorolac, surgical outcomes

## Abstract

**Background/Objectives**: Ketorolac is commonly used for pain management after orthopedic surgery, but concerns regarding its effects on postoperative complications remain. This study evaluates the impact of ketorolac use on short- and long-term outcomes in adult patients undergoing uncemented primary total hip arthroplasty (THA), where implant stability relies on biological fixation through bone ingrowth into a porous-coated prosthesis rather than bone cement. **Methods**: A retrospective cohort study was conducted using the TriNetX Research Network. Patients aged 18 years or older who underwent uncemented primary THA between 1 January 2004 and 1 January 2024 were included. Two cohorts were compared: those who received ketorolac on the day of or within one week of surgery and those who did not. Cohorts were propensity score-matched. Outcomes were assessed at 30 days, 1 year, and 5 years postoperatively. **Results**: At 30 days, ketorolac use was associated with significantly lower risks of transfusion (RR: 0.6, *p* < 0.01). However, it was linked to higher rates of acute posthemorrhagic anemia (RR: 1.2, *p* < 0.01) and periprosthetic fracture (RR: 1.4, *p* < 0.01). At 1 year, ketorolac use was associated with reduced risks of death (RR: 0.8, *p* < 0.01) and transfusion (RR: 0.7, *p* < 0.01), but increased risks of acute posthemorrhagic anemia (RR: 1.2, *p* < 0.01), deep surgical site infection (SSI) (RR: 1.8, *p* = 0.01), superficial SSI (RR: 1.9, *p* < 0.01), periprosthetic joint infection (RR: 1.1, *p* < 0.01), wound dehiscence (RR: 1.2, *p* < 0.01), periprosthetic mechanical complication (RR: 1.2, *p* < 0.01), and periprosthetic fracture (RR: 1.5, *p* < 0.01). **Conclusions**: Our findings highlight the complex risk profile of ketorolac in uncemented THA patients and suggest that clinicians should carefully consider individual patient factors and engage in shared decision-making when counseling patients on the use of ketorolac in the perioperative setting.

## 1. Introduction

Total hip arthroplasty (THA) is a widely performed procedure and within the field of arthroplasty, research is increasingly focused on optimizing perioperative management to reduce complications including cardiovascular events, thromboembolism, infections, and mechanical failures, which significantly impact patient morbidity and healthcare costs [1,2]. Medications commonly used in the perioperative period, including nonsteroidal anti-inflammatory drugs (NSAIDs), play a critical role in this optimization and warrant close examination.

Several studies have examined the role of NSAIDs in orthopedic procedures, with conflicting findings on their impact on wound healing, infection risk, and bone integration [3,4,5,6,7,8]. While ketorolac (Toradol) is effective for opioid sparing postoperative pain control, concerns about increased bleeding risk, renal impairment, and potential impacts on bone healing have led to caution in its routine use [3,9,10]. This caution is particularly important for uncemented THA, where implant stability relies on biological fixation through bone ingrowth into a porous-coated prosthesis rather than bone cement [11]. Since long-term success depends on effective osseous integration, factors that may influence osseous healing, such as NSAID use, must be carefully considered [12,13].

Given the potential for NSAIDs, such as ketorolac, to interfere with bone healing, it is imperative to better understand their role in perioperative management. Therefore, we asked: what are the perioperative outcomes associated with ketorolac use in uncemented THA? This study uniquely focuses on uncemented THA, where concerns about osseous integration are especially relevant, and aims to provide clarity on ketorolac’s safety profile in this specific surgical context. By addressing a gap in existing literature, our findings may inform more targeted perioperative analgesia protocols in arthroplasty.

## 2. Materials and Methods

### 2.1. Data Source

The TriNetX Research network (https://trinetx.com, Cambridge, MA, USA, access date: 1 April 2025) was utilized for this retrospective, propensity-matched study. The Research network contains one of the largest repositories of data from the United States, Canada, and Western Europe, encompassing inpatient, outpatient, emergency room, and other non-hospital facilities data, sourced from over 100 healthcare organizations (HCOs) and spanning more than 130 million patient records. Patient data from HCOs are derived from electronic health records (EHRs) and supplemented with information from over 100 commercial and government payers, including Medicare and Medicaid [14].

### 2.2. Patient Population

Patients greater than or equal to 18 years of age who underwent uncemented THA between 1 January 2004 and 1 January 2024 met the inclusion criteria. Exclusion criteria included patients less than 18 years of age. Two distinct cohorts were compared and analyzed: (1) patients who received ketorolac on the day of or within 1 week of their THA (+ketorolac) and, (2) patients who did not receive ketorolac on the day of or within 1 week of their THA (-ketorolac). Patient identification utilized appropriate CPT (Current Procedural Terminology), ICD-9 (International Classification of Diseases, 9th Revision) and ICD-10 (International Classification of Diseases, 10th Revision) codes relating to uncemented THA. All codes utilized in our analysis are provided in Appendix A.

### 2.3. Demographics

Patient demographic data obtained from the TriNetX Research network included age at index procedure, sex, race/ethnicity, and body mass index (BMI). In addition, baseline comorbidities were extracted, including diabetes mellitus, primary hypertension, hyperlipidemia, chronic ischemic heart disease, chronic lower respiratory disease, and tobacco use. Renal function was assessed using estimated glomerular filtration rate (eGFR) stratified by standard clinical categories. These demographic and clinical variables were used for both cohort description and propensity score matching to control for confounding.

### 2.4. Index Event and Outcome Analysis

The index event was defined as the date of primary uncemented THA. Outcomes were analyzed at 30 days, 1 year, and 5 years to evaluate both short-term and long-term complications. Cardiac outcomes including myocardial infarction (MI), cardiac arrest, and death were assessed. Renal and hematologic outcomes such as acute renal failure (ARF), transfusion requirements, pulmonary embolism (PE), lower extremity deep vein thrombosis (DVT), acute posthemorrhagic anemia, and hematoma were also analyzed. Infectious consequences, specifically deep and superficial SSI and periprosthetic joint infection (PJI) were assessed as well. Additionally, surgical/mechanical complications such as wound dehiscence, periprosthetic mechanical complications (i.e., abnormal movement, poor alignment), periprosthetic dislocation, periprosthetic fracture, and revision surgery were explored. A subgroup analysis was conducted to assess ARF and revision surgery rates in high-risk populations, specifically in patients over 75 years with diabetes mellitus and those over 70 with BMI > 25 kg/m^2^. Additional details on outcome definitions and coding criteria are provided in Appendix A.

### 2.5. Statistical Tools, Data Analysis, and Propensity Score Matching

Relative risk (RR), supplemented by absolute risk (AR), were used to compare the frequency of complications between the exposure and comparison groups, with 95% confidence intervals (Cis) provided for all relative risk calculations. The statistical tests used included Fisher’s exact test, Chi-square, and Student’s *t*-test. Statistical significance was established at *p* < 0.05 with two-sided tests. The TriNetX Live platform was used for data compilation. Microsoft Excel (2023) was utilized for further analysis and data visualization. The analytical procedures were verified independently by all co-authors and further confirmed by the corresponding author.

Patients in the +ketorolac and -ketorolac cohorts were subjected to matching based on age at index, sex and relevant comorbidities including tobacco use, diabetes mellitus, primary hypertension, hyperlipidemia, chronic ischemic heart disease, chronic lower respiratory disease, eGFR, and BMI, using a 1:1 propensity score matching algorithm.

The covariates selected for propensity score matching were chosen based on their established clinical relevance as potential confounders influencing postoperative outcomes and ketorolac use, with particular emphasis on renal function due to observed baseline differences in eGFR between groups. Incorporating eGFR into the matching process aimed to balance renal function across cohorts to minimize confounding bias.

## 3. Results

### 3.1. Demographic Characteristics

Before propensity score matching, 51,297 (40%) patients were in the +ketorolac cohort and 78,268 (60%) in the -ketorolac cohort. Patients in the +ketorolac cohort were significantly younger (62.5 years ± 11.4 vs. 63.2 years ± 11.2, *p* < 0.01) and had similar sex distribution (53% vs. 53% women, *p* = 0.05). They were also more likely to be Hispanic (3% vs. 2%, *p* < 0.01) or White (80% vs. 75%, *p* < 0.01) and less likely to be Asian (3% vs. 5%, *p* < 0.01). The +ketorolac group had a slightly higher prevalence of tobacco use (3% vs. 2%, *p* < 0.01), primary hypertension (43% vs. 42%, *p* < 0.01), hyperlipidemia (36% vs. 34%, *p* < 0.01), and chronic lower respiratory disease (15% vs. 14%, *p* < 0.01), while diabetes mellitus was similar (13% vs. 13%, *p* < 0.01). Renal function also differed between cohorts, with the +ketorolac group demonstrating higher proportions of preserved eGFR (≥90 mL/min/1.73 m^2^: 41% vs. 34%, *p* < 0.01) and lower proportions across all lower eGFR strata (*p* < 0.01).

After propensity score matching, 51,050 patients remained in each cohort, with no significant differences in sex distribution, tobacco use, or eGFR. Minor differences persisted in age (62.6 ± 11.4 vs. 62.4 ± 11.3, *p* = 0.03) and comorbidities, though all were marginal. Comprehensive patient demographic characteristics before and after matching are shown in Table 1 and Table 2, respectively.

### 3.2. Thirty-Day Outcomes

At 30 days, cardiovascular outcomes were similar between groups, with no significant difference in MI (RR: 0.9, *p* = 0.60), cardiac arrest (RR: 1.5, *p* = 0.17), or death (RR: 0.8, *p* = 0.29). Among renal, vascular, and hematologic outcomes, +ketorolac was associated with a significantly lower risk of transfusion (RR: 0.6, *p* < 0.01), but a higher risk of acute posthemorrhagic anemia (RR: 1.2, *p* < 0.01). There were no significant differences in PE, DVT, hematoma, or ARF. Among infectious outcomes, +ketorolac was associated with no significant differences in deep SSI, superficial SSI, or PJI. For surgical and mechanical outcomes, +ketorolac was associated with a higher risk of periprosthetic fracture (RR: 1.4, *p* < 0.01), with no significant differences in wound dehiscence, periprosthetic mechanical complications, dislocation, or revision surgery. Comprehensive 30-day outcomes are shown in Table 3.

### 3.3. One-Year Outcomes

At one year, there remained no significant differences in MI (RR: 1.0, *p* = 0.82) or cardiac arrest (RR: 1.0, *p* = 0.94), but mortality was significantly lower in the +ketorolac group (RR: 0.8, *p* < 0.01). Ketorolac use was also associated with a significantly lower risk of transfusion (RR: 0.7, *p* < 0.01), and a higher risk of acute posthemorrhagic anemia (RR 1.2, *p* < 0.01). There were no differences in DVT, PE, hematoma, or ARF. In terms of infectious outcomes, both deep SSI (RR: 1.8, *p* = 0.01) and superficial SSI (RR: 1.9, *p* < 0.01) were significantly higher in the ketorolac cohort, as was the risk of PJI (RR: 1.1, *p* < 0.01). Surgical complications were more frequent in +ketorolac cohort, including increased wound dehiscence (RR: 1.2, *p* < 0.01), periprosthetic mechanical complications (RR: 1.2, *p* < 0.01), and periprosthetic fracture (RR: 1.5, *p* < 0.01), though revision surgery was not significantly different (RR: 1.0, *p* = 0.69). Comprehensive 1-year outcomes are shown in Table 4.

### 3.4. Five-Year Outcomes

At five years, the +ketorolac group exhibited no significant difference in the risk of revision surgery (RR: 1.0, *p* < 0.54) (Table 4).

### 3.5. Subgroup Analysis: Renal and Revision Surgery Outcomes in High-Risk Populations

Among patients older than 75 with diabetes mellitus, the risk of 30-day ARF was not significantly different between +ketorolac and -ketorolac groups. In patients over 70 with BMI > 25 kg/m^2^, the risk of revision surgery at five years was significantly lower in the +ketorolac group (RR: 0.8, *p* < 0.01). Comprehensive subgroup analysis outcomes are shown in Table 4.

## 4. Discussion

The use of NSAIDs, such as ketorolac, in orthopedic surgeries has raised concerns about their impact on postoperative outcomes, including bone healing, cardiovascular and renal health [3,9,15]. This study aimed to assess ketorolac’s effects on mortality, cardiovascular function, infections, and surgical outcomes in uncemented THA patients. Our study found that patients in the +ketorolac group experienced lower mortality and transfusion rates, and higher risk of acute posthemorrhagic anemia, superficial SSI, deep SSI, PJI, periprosthetic fracture, wound dehiscence, and mechanical complications. There were no significant differences in MI, cardiac arrest, PE, DVT, ARF, or revision surgery at any time point.

Our study found no significant differences in MI or cardiac arrest at 30 days or one year between patients who received ketorolac and those who did not. However, at the one-year mark, ketorolac use was associated with a significantly lower risk of mortality (RR 0.8, *p* < 0.01). This finding contrasts with prior studies that have raised concerns about NSAID use and increased cardiovascular risk [16,17]. One possible explanation for the observed mortality benefit is ketorolac’s opioid-sparing effect, which may reduce opioid-related adverse events such as respiratory depression [18,19]. While the 30-day mortality difference was not statistically significant, the reduction in mortality at one year suggests a possible long-term protective effect. Baseline characteristics showed a higher proportion of patients with preserved renal function in the ketorolac group, which may have influenced outcomes. Although propensity score matching and adjustment for known confounders were performed, residual confounding or unmeasured factors cannot be entirely ruled out as contributors to the observed associations. Further research is warranted to clarify whether ketorolac’s mortality benefit is driven by improved perioperative recovery or reflects confounding by indication.

Our analysis of renal and hematologic outcomes revealed that ketorolac was associated with a similar risk of ARF at 30 days and 1-year follow-up, alongside a significantly lower risk of transfusion at both time points. However, ketorolac was also linked to an increased risk of acute posthemorrhagic anemia (RR 1.2, *p* < 0.01 at both 30 days and one year), highlighting a notable hematologic risk despite the reduced transfusion rates. These findings contrast somewhat with prior literature suggesting ketorolac may increase the risk of acute kidney injury (AKI), particularly in patients with preexisting renal impairment [9]. Although propensity score matching balanced baseline eGFR between groups, ketorolac recipients had better renal function before matching (31% vs. 37% with eGFR <60 mL/min/1.73 m^2^), likely reflecting clinical prescribing patterns. Thus, residual confounding may persist, and it is possible that improved baseline renal reserve among ketorolac recipients contributed to the observed lack of increased ARF risk despite the drug’s known nephrotoxic potential. Additionally, our findings of reduced transfusion risk are also unexpected, given ketorolac’s platelet inhibitory effects and potential to increase bleeding risk [20,21]. The paradox of a lower transfusion risk despite a higher incidence of acute posthemorrhagic anemia may be explained by several factors. First, the anemia observed could be primarily mild or subclinical, insufficient to trigger transfusions but still detectable through laboratory measures. Second, ketorolac’s use may have been limited to patients with better baseline hemostatic profiles and less severe bleeding risk, leading to fewer clinically significant hemorrhagic events requiring transfusion. Third, ketorolac’s anti-inflammatory effects may reduce overall surgical blood loss and postoperative inflammation, thereby lowering the need for transfusions even in the presence of anemia. Lastly, institutional transfusion thresholds and clinical decision-making may also contribute, as providers might manage anemia conservatively without transfusions when possible. Together, these factors suggest that while ketorolac increases the risk of posthemorrhagic anemia, this does not necessarily translate into a proportionate increase in transfusion requirements. Further studies are needed to determine whether these associations hold in broader patient populations.

Infectious outcomes showed a complex pattern. At 30 days, there were no significant differences in superficial SSI, deep SSI, or PJI. However, by one year, ketorolac was associated with significantly higher risks of both superficial (RR 1.9, *p* < 0.01) and deep SSI (RR 1.8, *p* = 0.01), as well as increased PJI risk (RR 1.1, *p* < 0.01). These findings align with prior research suggesting that NSAIDs may impair immune function and hinder tissue healing by inhibiting prostaglandin synthesis, which plays a role in the inflammatory response necessary for tissue repair [22,23]. Studies have shown that NSAID use can lead to reduced angiogenesis, fibroblast proliferation, and collagen deposition, all of which are critical for proper wound healing [24,25,26]. The combination of impaired tissue healing and a weakened immune response may explain the higher risk of superficial and deep SSI. The delayed divergence in infection rates suggests that ketorolac may not compromise immediate wound healing but may negatively affect the later phases of tissue repair and host defense. Further studies are needed to evaluate the overall effect of ketorolac on SSIs and determine whether alternative dosing strategies or adjunctive therapies could mitigate the aforementioned risks.

Regarding surgical and mechanical outcomes, ketorolac use was associated with increased risk of periprosthetic fractures at 30 days (RR 1.4, *p* < 0.01) and one year (RR 1.5, *p* < 0.01), consistent with previous concerns that NSAIDs interfere with bone healing by reducing prostaglandin-mediated osteogenesis [3,15]. Additionally, at one year, ketorolac use was linked to higher rates of wound dehiscence (RR 1.2, *p* < 0.01) and periprosthetic mechanical complications (RR 1.2, *p* < 0.01), although revision surgery rates were not significantly different at any time point. However, a notable exception was found in a high-risk subgroup of patients over age 70 years with BMI > 25 kg/m^2^, suggesting that despite its effects on bone healing, ketorolac was associated with a lower 5-year revision risk (RR 0.8, *p* < 0.01). Despite the increased fracture risk, the lower revision surgery rate in high-risk patients suggests that other factors, such as reduced opioid-related complications, including impaired mobility, hyperalgesia, somnolence, immunosuppression, and delayed wound healing, may contribute to overall implant longevity [27,28]. However, the authors acknowledge this as a hypothesis-driven finding requiring further research to confirm the underlying mechanisms and to clarify the balance of risks and benefits of ketorolac use in different patient populations. These findings highlight the complexity of balancing the analgesic benefits of ketorolac with its potential effects on bone health, underscoring the need for patient-specific risk stratification when considering perioperative NSAID use in THA.

### Limitations

While our study has several strengths, including its large sample size, comprehensive outcome assessment, and rigorous statistical methodology, certain limitations must be acknowledged. As a retrospective study utilizing the TriNetX database, there is an inherent risk of selection bias and residual confounding despite the use of propensity score matching. Additionally, because the TriNetX database is a voluntary data-sharing platform, large academic medical centers may be overrepresented, potentially limiting the generalizability of our findings to broader healthcare settings. The study also relies on administrative coding for patient identification and outcome classification, introducing the possibility of coding errors or misclassification.

Another key limitation is the lack of detailed, patient-level data on ketorolac dosing, route of administration, and frequency of use. Without this information, we are unable to assess potential dose-dependent effects on postoperative complications, particularly its impact on bone healing, infection risk, and renal function. Furthermore, we do not have access to the specific indications for revision surgery. As a result, we cannot determine whether revisions were performed for implant subsidence, which would be particularly relevant in evaluating osseointegration in uncemented THA. This limits our ability to assess the relationship between ketorolac use and mechanical failure due to impaired bone ingrowth. Greater clarity in the revision surgery category is needed to support conclusions about implant performance. To this end, we were unable to directly assess opioid-related adverse events or other factors that may explain the observed reduction in 1-year mortality in the ketorolac group, introducing the potential for confounding by indication.

Additionally, we did not collect data on bone mineral density and osteoporosis treatment, which may influence uncemented implant fixation and postoperative outcomes. Furthermore, the lack of detailed information on anemia severity and transfusion thresholds limits our ability to fully interpret the observed discrepancy between reduced transfusion rates and increased acute posthemorrhagic anemia in the ketorolac group. A further limitation is the absence of data on important confounding factors such as operative time, antibiotic prophylaxis protocols, and glycemic control, which are known to influence infection risk and limit the ability to draw definitive causal conclusions regarding the association between ketorolac use and increased postoperative infections. Finally, we were unable to evaluate patient-reported outcomes, such as pain relief, functional recovery, or quality of life, which are essential in assessing the true clinical impact of ketorolac use following joint arthroplasty. Future research incorporating detailed pharmacologic data and patient-centered outcomes will be necessary to provide a more comprehensive understanding of the risks and benefits associated with ketorolac in this surgical population.

## 5. Conclusions

Ketorolac use in uncemented THA was associated with a reduced risk of death and transfusion, but a higher risk of acute posthemorrhagic anemia, superficial and deep SSI, PJI, wound dehiscence, and periprosthetic mechanical and fracture complications. Cardiovascular and thromboembolic complications did not differ significantly between groups. These findings highlight the complex risk profile of ketorolac in uncemented THA patients and suggest that clinicians should carefully consider individual patient factors and engage in shared decision-making when counseling patients on the use of ketorolac in the perioperative setting.

## Figures and Tables

**Table 1:** Patient demographic characteristics before match.

	+Ketorolac	−Ketorolac	
Characteristic	N (Mean or %)	N (Mean or %)	*p*
Age at Index	51,297 (62.5 ± 11.4)	78,268 (63.2 ± 11.2)	<0.01
Sex			
Men	23,187 (45%)	35,321 (45%)	0.80
Women	27,303 (53%)	41,218 (53%)	0.05
Race and Ethnicity			
Hispanic or Latino	1637 (3%)	1557 (2%)	<0.01
Asian	1439 (3%)	3524 (5%)	<0.01
Black or African American	4871 (9%)	7293 (9%)	0.28
White	41,253 (80%)	58,868 (75%)	<0.01
Other Race	895 (2%)	1157 (1%)	<0.01
Diagnosis			
Tobacco Use	1662 (3%)	1596 (2%)	<0.01
Diabetes Mellitus	6429 (13%)	10,447 (13%)	<0.01
Primary Hypertension	22,292 (43%)	32,829 (42%)	<0.01
Hyperlipidemia	18,217 (36%)	26,895 (34%)	<0.01
Chronic Ischemic Heart Disease	5072 (10%)	8144 (10%)	<0.01
Chronic Lower Respiratory Disease	7644 (15%)	11,035 (14%)	<0.01
Body Mass Index			
Mean Body Mass Index	30.1 ± 6.37	29.9 ± 6.37	<0.01
eGFR			
At least 90 mL/min/1.73 m^2^	21,283 (41%)	26,724 (34%)	<0.01
60–90 mL/min/1.73 m^2^	30,918 (60%)	42,865 (55%)	<0.01
45–60 mL/min/1.73 m^2^	10,540 (21%)	17,026 (22%)	<0.01
30–45 mL/min/1.73 m^2^	3658 (7%)	7273 (9%)	<0.01
15–30 mL/min/1.73 m^2^	1067 (2%)	2641 (3%)	<0.01
At most 15 mL/min/1.73 m^2^	456 (1%)	2067 (3%)	<0.01

**Table 2:** Patient demographic characteristics after match.

	+Ketorolac	−Ketorolac	
Characteristic	N (Mean or %)	N (Mean or %)	*p*
Age at Index	51,050 (62.6 ± 11.4)	51,050 (62.4 ± 11.3)	0.03
Sex			
Men	23,066 (45%)	23,130 (45%)	0.69
Women	27,177 (53%)	27,130 (53%)	0.77
Race and Ethnicity			
Hispanic or Latino	1631 (3%)	1088 (2%)	<0.01
Asian	1438 (3%)	2439 (5%)	<0.01
Black or African American	4827 (9%)	4812 (9%)	0.87
White	41,064 (80%)	38,666 (76%)	<0.01
Other Race	890 (2%)	778 (2%)	<0.01
Diagnosis			
Tobacco Use	1421 (3%)	1391 (3%)	0.57
Diabetes Mellitus	6396 (13%)	5984 (12%)	<0.01
Primary Hypertension	22,120 (43%)	21,432 (42%)	<0.01
Hyperlipidemia	18,074 (35%)	17,452 (34%)	<0.01
Chronic Ischemic Heart Disease	5035 (10%)	4716 (9%)	<0.01
Chronic Lower Respiratory Disease	7534 (15%)	7084 (14%)	<0.01
Body Mass Index			
Mean Body Mass Index	30.1 ± 6.37	30 ± 6.28	0.05
eGFR			
At least 90 mL/min/1.73 m^2^	21,055 (41%)	20,926 (41%)	0.41
60–90 mL/min/1.73 m^2^	30,702 (60%)	30,711 (60%)	0.95
45–60 mL/min/1.73 m^2^	10,475 (21%)	10,095 (20%)	<0.01
30–45 mL/min/1.73 m^2^	3653 (7%)	3330 (7%)	<0.01
15–30 mL/min/1.73 m^2^	1067 (2%)	1023 (2%)	0.33
At most 15 mL/min/1.73 m^2^	456 (1%)	496 (1%)	0.19

**Table 3:** Thirty-day outcomes.

Measure	+Ketorolac Proportion	−Ketorolac Proportion	Risk Ratio	95% CI	*p*
Cardiovascular Outcomes					
Myocardial Infarction	0.3%	0.3%	0.9	(0.7, 1.2)	0.60
Cardiac Arrest	0.1%	0.1%	1.5	(0.9, 2.4)	0.17
Death	0.1%	0.2%	0.8	(0.6, 1.1)	0.29
Renal, Vascular and Hematologic Outcomes					
Transfusion	2.7%	4.4%	0.6	(0.6, 0.7)	<0.01
Pulmonary Embolism	0.6%	0.5%	1.2	(1.0, 1.4)	0.11
Deep Vein Thrombosis (Lower Extremity)	0.9%	0.9%	0.9	(0.8, 1.1)	0.41
Acute Posthemorrhagic Anemia	7.7%	6.6%	1.2	(1.1, 1.2)	<0.01
Hematoma	0.0%	0.0%	1.0	(0.6, 1.7)	1.00
Acute Renal Failure	1.3%	1.2%	1.1	(1.0, 1.2)	0.29
Infectious Outcomes					
Deep Surgical Site Infection	0.0%	0.0%	1.9	(0.9, 4.0)	0.11
Superficial Surgical Site Infection	0.1%	0.1%	1.5	(1.0, 2.3)	0.05
Periprosthetic Joint Infection	1.4%	1.3%	1.1	(1.0, 1.2)	0.07
Surgical and Mechanical Outcomes					
Wound Dehiscence	0.5%	0.4%	1.2	(1.0, 1.5)	0.06
Periprosthetic Mechanical Complication	0.4%	0.4%	1.1	(0.9, 1.3)	0.45
Periprosthetic Dislocation	0.9%	0.8%	1.1	(1.0, 1.2)	0.24
Periprosthetic Fracture	1.1%	0.8%	1.4	(1.2, 1.6)	<0.01
Revision Surgery	0.7%	0.7%	1.0	(0.9, 1.2)	0.94

**Table 4:** One-year, five-year, and subgroup analysis outcomes.

Measure	+Ketorolac Proportion	−Ketorolac Proportion	Risk Ratio	95% CI	*p*
Cardiovascular Outcomes					
Myocardial Infarction	0.9%	0.9%	1.0	(0.9, 1.1)	0.82
Cardiac Arrest	0.2%	0.2%	1.0	(0.8, 1.3)	0.94
Death	0.8%	1.0%	0.8	(0.7, 0.9)	<0.01
Renal, Vascular and Hematologic Outcomes					
Transfusion	3.7%	5.5%	0.7	(0.6, 0.7)	<0.01
Pulmonary Embolism	1.2%	1.1%	1.1	(1.0, 1.2)	0.21
Deep Vein Thrombosis (Lower Extremity)	2.0%	2.0%	1.0	(0.9, 1.1)	0.70
Acute Posthemorrhagic Anemia	10.3%	8.8%	1.2	(1.1, 1.2)	<0.01
Hematoma	0.1%	0.1%	1.0	(0.7, 1.4)	0.93
Acute Renal Failure	2.9%	2.7%	1.1	(1.0, 1.2)	0.06
Infectious Outcomes					
Deep Superficial Surgical Site Infection	0.1%	0.1%	1.8	(1.2, 2.7)	0.01
Superficial Surgical Site Infection	0.3%	0.2%	1.9	(1.5, 2.4)	<0.01
Periprosthetic Joint Infection	2.8%	2.5%	1.1	(1.0, 1.2)	<0.01
Surgical and Mechanical Outcomes					
Wound Dehiscence	1.1%	0.9%	1.2	(1.1, 1.4)	<0.01
Periprosthetic Mechanical Complication	1.2%	1.0%	1.2	(1.1, 1.4)	<0.01
Periprosthetic Dislocation	2.0%	1.9%	1.1	(1.0, 1.2)	0.12
Periprosthetic Fracture	2.4%	1.6%	1.5	(1.4, 1.6)	<0.01
Revision Surgery	1.9%	1.9%	1.0	(0.9, 1.1)	0.69
Subgroup Analysis					
30-Day Renal Failure (Patients > 75 with Diabetes Mellitus)	5.7%	5.5%	1.0	(0.7, 1.4)	0.93
1-Year Revision Surgery (Patients > 70 and Body Mass Index > 25)	1.7%	2.0%	0.9	(0.7, 1.1)	0.21
5-Year Revision Surgery (Patients > 70 and Body Mass Index > 25)	4.9%	5.8%	0.8	(0.7, 1.0)	0.01
5-Year Revision Surgery	2.9%	3.0%	1.0	(0.9, 1.0)	0.54

## Data Availability

The data presented in this study are available on reasonable request from the corresponding author.

## Abbreviations

The following abbreviations are used in this manuscript:

THA	Total Hip Arthroplasty
RR	Risk Ratio
SSI	Surgical Site Infection
NSAID	Nonsteroidal Anti-Inflammatory
HCO	Healthcare Organization
HER	Electronic Health Record
CPT	Current Procedural Terminology
ICD-9	International Classification of Diseases, 9th Revision
ICD-10	International Classification of Diseases, 10th Revision
eGFR	Estimated Glomerular Filtration Rate
MI	Myocardial Infarction
ARF	Acute Renal Failure
PE	Pulmonary Embolism
DVT	Deep Vein Thrombosis
PJI	Periprosthetic Joint Infection
AR	Absolute Risk
Ci	Confidence Interval
HIPAA	Health Insurance Portability and Accountability Act
IRB	Institutional Review Board

## Appendix A

**Table A1:** Patient Population Codes.

Code	Description
UMLS:ICD10PCS:0SR90JA	Replacement of Right Hip Joint with Synthetic Substitute, Uncemented, Open Approach
UMLS:ICD10PCS:0SRB0JA	Replacement of Left Hip Joint with Synthetic Substitute, Uncemented, Open Approach
UMLS:ICD10PCS:0SR902A	Replacement of Right Hip Joint with Metal on Polyethylene Synthetic Substitute, Uncemented, Open Approach
UMLS:ICD10PCS:0SR903A	Replacement of Right Hip Joint with Ceramic Synthetic Substitute, Uncemented, Open Approach
UMLS:ICD10PCS:0SR901A	Replacement of Right Hip Joint with Metal Synthetic Substitute, Uncemented, Open Approach
UMLS:ICD10PCS:0SR904A	Replacement of Right Hip Joint with Ceramic on Polyethylene Synthetic Substitute, Uncemented, Open Approach
UMLS:ICD10PCS:0SR906A	Replacement of Right Hip Joint with Oxidized Zirconium on Polyethylene Synthetic Substitute, Uncemented, Open Approach
UMLS:ICD10PCS:0SRB06A	Replacement of Left Hip Joint with Oxidized Zirconium on Polyethylene Synthetic Substitute, Uncemented, Open Approach
UMLS:ICD10PCS:0SRB01A	Replacement of Left Hip Joint with Metal Synthetic Substitute, Uncemented, Open Approach
UMLS:ICD10PCS:0SRB02A	Replacement of Left Hip Joint with Metal on Polyethylene Synthetic Substitute, Uncemented, Open Approach
UMLS:ICD10PCS:0SRB03A	Replacement of Left Hip Joint with Ceramic Synthetic Substitute, Uncemented, Open Approach
UMLS:ICD10PCS:0SRB04A	Replacement of Left Hip Joint with Ceramic on Polyethylene Synthetic Substitute, Uncemented, Open Approach
NLM:RXNORM:35827	ketorolac

**Table A2:** Outcome Codes.

Code	Description
UMLS:ICD10PCS:30233N1	Transfusion
UMLS:ICD10CM:I21	Myocardial Infarction
UMLS:ICD10CM:I26	Pulmonary Embolism
UMLS:ICD10CM:I82.4	Deep Vein Thrombosis
UMLS:ICD10CM:M79.81	Hematoma
UMLS:ICD10CM:T84.5	Periprosthetic Joint Infection
UMLS:ICD10CM:N17	Acute Renal Failure
UMLS:ICD10CM:D62	Acute Posthemorrhagic Anemia
UMLS:ICD10CM:T81.30XA; UMLS:ICD10CM:T81.31XA; UMLS:ICD10CM:T81.32XA.	Wound Dehiscence
UMLS:ICD10CM:T81.42	Deep SSI
UMLS:ICD10CM:T81.41	Superficial SSI
UMLS:ICD10CM:T84.09	Periprosthetic Mechanical Complication
UMLS:ICD10CM:T84.02	Periprosthetic Dislocation
UMLS:ICD10CM:T84.01; UMLS:ICD10CM:M96.6; UMLS:ICD10CM:M97.0	Periprosthetic Fracture
UMLS:ICD10CM:I46; or UMLS:ICD10CM:I97.12.	Cardiac Arrest
